# Protective Effect of Antioxidants in Nitric Oxide/COX-2 Interaction during Inflammatory Pain: The Role of Nitration

**DOI:** 10.3390/antiox9121284

**Published:** 2020-12-16

**Authors:** Sara Ilari, Concetta Dagostino, Valentina Malafoglia, Filomena Lauro, Luigino Antonio Giancotti, Antonella Spila, Stefania Proietti, Domenica Ventrice, Milena Rizzo, Micaela Gliozzi, Ernesto Palma, Fiorella Guadagni, Daniela Salvemini, Vincenzo Mollace, Carolina Muscoli

**Affiliations:** 1 Institute of Research for Food Safety & Health (IRC-FSH), Department of Health Science, University “Magna Graecia” of Catanzaro, 88100 Catanzaro, Italy; sara.ilari@hotmail.it (S.I.); c_dagostino@libero.it (C.D.); micaela.gliozzi@gmail.com (M.G.); palma@unicz.it (E.P.); mollace@libero.it (V.M.); 2 Institute for Research on Pain, ISAL Foundation, 47922 Torre Pedrera, Italy; valentinamalafoglia@yahoo.it; 3 Department of Pharmacology and Physiology, Henry and Amelia Nasrallah Center for Neuroscience, Saint Louis University School of Medicine, St. Louis, MO 63104, USA; milena.lauro@health.slu.edu (F.L.); luigi.giancotti@health.slu.edu (L.A.G.); daniela.salvemini@health.slu.edu (D.S.); 4 Department of Human Science & Quality of Life Promotion, San Raffaele Roma Open University, via di Val Cannuta 247, 0166 Rome, Italy; antonella.spila@sanraffaele.it; 5 Scientific Direction, IRCCS San Raffaele Pisana, via di Val Cannuta 247, 0166 Rome, Italy; stefania.proietti@sanraffaele.it; 6 ARPACAL, 88100 Catanzaro, Italy; d.ventrice@arpacal.it; 7 Department of Drug Science, University of Catania, Viale Andrea Doria 6, 95125 Catania, Italy; milena.rizzo@unict.it; 8 Department of Human Science & Quality of Life Promotion, SanRaffaele Roma Open University, and Inter-Institutional Multidisciplinary BioBank (BioBIM), IRCCS San Raffaele Pisana, via di Val Cannuta 247, 0166 Rome, Italy; fiorella.guadagni@sanraffaele.it

**Keywords:** inflammation, cyclooxygenase 2 (COX-2), prostaglandin E_2_ (PGE_2_), lactate dehydrogenase (LDH), nitration, malondialdehyde (MDA), reactive oxygen species (ROS), reactive nitrogen species (RNS), antioxidants

## Abstract

In clinical practice, inflammatory pain is an important, unresolved health problem, despite the utilization of non-steroidal anti-inflammatory drugs (NSAIDs). In the last decade, different studies have proven that reactive oxygen species (ROS) and reactive nitrogen species (RNS) are involved in the development and maintenance of inflammatory pain and hyperalgesia via the post-translation modification of key proteins, such as manganese superoxide dismutase (MnSOD). It is well-known that inducible cyclooxygenase 2 (COX-2) plays a crucial role at the beginning of the inflammatory response by converting arachidonic acid into proinflammatory prostaglandin PGE_2_ and then producing other proinflammatory chemokines and cytokines. Here, we investigated the impact of oxidative stress on COX-2 and prostaglandin (PG) pathways in paw exudates, and we studied how this mechanism can be reversed by using antioxidants during hyperalgesia in a well-characterized model of inflammatory pain in rats. Our results reveal that during the inflammatory state, induced by intraplantar administration of carrageenan, the increase of PGE_2_ levels released in the paw exudates were associated with COX-2 nitration. Moreover, we showed that the inhibition of ROS with Mn (III) tetrakis (4-benzoic acid) porphyrin(MnTBAP) antioxidant prevented COX-2 nitration, restored the PGE_2_ levels, and blocked the development of thermal hyperalgesia.

## 1. Introduction

Inflammatory pain is considered a major health issue. Nowadays, non-steroidal anti-inflammatory drugs (NSAIDs) are the most common class of analgesics for moderate/severe inflammatory pain—in particular, non-selective cyclooxygenase 1/cyclooxygenase 2 (COX-1/COX-2) or more selective COX-2 inhibitors are the mostly used formulations, even if various side effects associated with their prolonged utilization are well-documented [1,2,3,4].

During acute/chronic inflammation, hyperalgesia is the result of a persistent state of peripheral afferent sensitization, which subsequently leads to spinal sensitization through the release of the excitatory amino acid glutamate and free radical production (reactive oxygen species (ROS) and reactive nitrogen species (RNS)) [5,6,7,8].

Free radical oxidative damage is strictly linked with oxidative stress, involved in several degenerative diseases, such as inflammatory, cardiovascular, and digestive diseases, as well as cancer, aging, and stroke [9,10,11,12,13].

Free radical activity includes many proinflammatory effects—for example, endothelial cell damage and increased microvascular permeability, the recruitment of neutrophils at the sites of inflammation [14], the autocatalytic destruction of neurotransmitters and hormones (e.g., noradrenaline and adrenaline), lipid peroxidation and oxidation, DNA damage, and activation of poly-ADP-ribose polymerase (PARP) [15,16,17,18,19].

The concentration of nitroxidative species (ROS/RNS) can increase during stress conditions, inducing the activation of transcription factors (e.g., activator protein 1 (AP1) and nuclear factor kappa (NF-kB)) and mitogen-activated protein kinases (MAPKs), which can in turn activate COX enzymes and increase the production of prostaglandins (PGs) [10,20,21].

Consequently, prostaglandins, through the activation of different nociceptors, lead to an increase of phospholipase A_2_ (PLA_2_), and then of arachidonic acid (AA) levels [12,22,23].

Moreover, nitric oxide (NO) seems to interfere directly with the activity of COX-2, and therefore with the production of PGs; the metabolites of the arachidonic acid, similarly, would be able to modulate the biosynthesis of NO [10,20,21,24], thus generating a vicious circle mechanism. Therefore, the generation of free radicals and PGs plays a crucial role, enhancing pain sensitivity experienced during inflammatory diseases [21,22].

Pharmacological removal of ROS/RNS by antioxidants [25,26,27,28] can prevent the characteristic findings associated with inflammatory pain and other different etiologies of pain [26,28,29]. In particular, our previous data have already demonstrated the ability of Mn (III) tetrakis (4-benzoic acid) porphyrin (MnTBAP; a peroxynitrite decomposition catalyst) to inactivate the development of thermal hyperalgesia and reduce malondialdehyde (MDA) and 4-hydroxynonenal (4-HNE) formation, as well as the post-translational modification of cellular proteins [9,28], thus restoring the activity of endogenous enzymes [28].

To date, the cross-talk mechanism through which free radicals and COXs pathway contribute to the development and maintenance of hyperalgesia needs to be better clarified [21]. In the present study, we investigated the impact of oxidative stress on COX-2 and PG pathways in paw exudates, and how this mechanism can be reversed by the utilization of widely employed antioxidants, such as MnTBAP, during hyperalgesia, in a well-characterized model of inflammatory pain in rats.

Our findings underline the pivotal role of nitroxidative stress in hyperalgesia pathway. Thus, we suggest that the chronicization of pain could depend on post-translational nitration of key enzymes and transporters linked to glutamatergic neuro-transmission.

## 2. Materials and Methods

### 2.1. Animals

Male Sprague–Dawley rats (225−250 g, 8 weeks old; Envigo) were used following the Italian regulations for the protection of animals used for experimental and other scientific purposes (D.L. 26/2014); the European Economic Community regulations (2010/63/UE), with authorization number 577-2016-PR; and the National Institutes of Health (NIH) guidelines on laboratory animal welfare. The numbers of animals used are the minimum number necessary to achieve statistical significance at *p* < 0.05, as set forth by the International Society for the Study of Pain guidelines [30]. Rats (two per cage) were housed and preserved at fixed temperature (21 ± 1 °C) and humidity (60% ± 5%) conditions, allowed food ad libitum, and in a 12 h light/12 h dark cycle. Experiments were performed between 7:00 and 10:00 a.m. in a quiet room. Unless specified, all drugs were purchased from Sigma Aldrich and dissolved in saline (sodium chloride 0.9%).

### 2.2. Experimental Groups

Rats were allocated into one of the following experimental groups:

- Vehicle group: animals (*n* = 12) received an intraperitoneal (i.p.) injection of saline 15 min before intraplantar (i.pl.) injection of saline into the hindpaw;
- Carr group (*n* = 12): 15 min before intraplantar injection of carrageenan (1% suspension in 0.85% NaCl; Calbiochem) into the right hindpaw, rats received an intraperitoneal saline injection;
- Drugs groups: animals (*n* = 12 for each dose) received an intraperitoneal injection of different doses of MnTBAP (5 mg/kg, 10 mg/kg, or 30 mg/kg) 15 min before intraplantar injection of carrageenan (1% suspension in 0.85% NaCl; Calbiochem) into the right hindpaw.

The dose and the timing of MnTBAP administration were chosen according to the literature [6,28,31]. For all groups, 6 h following the intraplantar injection of carrageenan, rats were sacrificed, and each paw was cut at the level of the calcaneus bone. The paws’ soft tissue was immediately frozen in liquid nitrogen and stored at −80 °C for subsequent analyses (Western blot, manganese superoxide dismutase (MnSOD) activity, MALDI mass spectra (MS) and MDA analysis). For lactate dehydrogenase (LDH) and prostaglandin E_2_ (PGE_2_) quantification, paw tissues were centrifuged at 250× *g* for 20 min, as previously described [1,32,33], and the oedematous fluid (exudate) was recovered and analyzed.

### 2.3. Measurements of Thermal Hyperalgesia and Oedema after Carrageenan Administration

Hyperalgesic responses were detected following Hargreaves’s protocol [34]. Twenty seconds of cut-off latency was used in order to prevent tissue damage in non-responsive animals. Single rats acclimatized in a plexiglass chamber for 30 min. A high-intensity projector bulb (mobile unit) was used to make a thermal stimulus directly to the hind paw beneath the chamber. A thermocouple and an electronic clock circuit were used to determine the withdrawal latency period of injected and controlateral paws, to the nearest 0.1 s. The test ended when the animal failed to respond by 20 s. Every single point represents the delta change (s) in withdrawal latency (withdrawal latency of controlateral (left paw) minus withdrawal latency of injected paw (right paw)) at each time point. The results are reported as paw withdrawal latency changes (s). Paw volume changes were measured as reported in the literature [33]. In brief, paw volume was quantified through a plethysmometer (Ugo-Basile, Varese, Italy) at 6 h after carrageenan injection. For each animal, the increase in paw volume (mL) after the injection of carrageenan relative to pre-injection values describes oedema values. Each histogram represents the change (mL) in withdrawal latency (withdrawal latency of injected paw (right paw) minus withdrawal latency of controlateral (left paw)). Results are represented as paw volume change (mL).

### 2.4. Determination of Prostaglandin E_2_ (PGE_2_) Levels in Paw Exudate

PGE_2_ released in the paw exudates was measured by enzyme-linked immunosorbent assay (ELISA), as described previously [1,21,32,33], using commercially available kits (Amersham). Briefly, paws were gently centrifuged at 250× *g* for 20 min in order to recover a sample of the oedematous fluid, and the volume of fluid recovered from each paw was measured. Results were expressed in pg/paw, normalizing values to the amount of exudates recovered from each paw. All determinations were performed in triplicate.

### 2.5. Determination of Lactate Dehydrogenase (LDH) Levels in Paw Exudate

Paw exudate, recovered through a centrifugation of 250× *g* for 20 min, was measured. The levels of LDH in the paw exudates (5 µL of a 1:10-fold dilution of each sample in saline) were used as an indicator of cell toxicity and cell death, and were spectrophotometrically measured (at an absorbance of 340 nm) using an LDH assay kit (Sigma). All determinations were performed in triplicate.

### 2.6. Evaluation of Malondialdehyde (MDA) Levels in Rats after Carrageenan Treatment

MDA quantification was measured in the paw soft tissues through a thiobarbituric acid (TBA) reactive substance (TBARS) assay, as previously described [26]. Tissue samples were added to a vial containing 10% NaOH, 20% acetic acid, and TBA, and were boiled at 95 °C. After 1 h, the tubes were placed on ice to stop the reaction. Before being transferred to a black 96-well microliter plate, samples were centrifugated 10 min at 1600× *g* at 4 °C. The MDA–TBA adduct was fluorometrically measured at an excitation wavelength of 530 nm and emission wavelength of 550 nm using an Infinite 200 microplate fluorometer (Tecan). All determinations were performed in triplicate.

### 2.7. Tissue Preparation for Cytosolic Extraction

Paw soft tissue homogenization was performed in lysis buffer (20 mM Tris-base, 150 mM NaCl, 10% glycerol, 0.1% Triton-X-100, 1% Chaps, and 2 mM Ethylene glycol tetraacetic acid (EGTA)) with the addition of 1% protease inhibitor cocktail (*v*/*v*). Extracts were sonicated for 5 min (Fisher Scientific Sonicator) and incubated for 10 min on ice, and then were centrifuged at 12,500× *g* for 30 min at 4 °C. The obtained supernatants were stored immediately at −80 °C and used to evaluate immunoprecipitation and for Western blot analyses. Protein concentrations were determined using the bicinchoninic acid (BCA) protein assay (Pierce).

### 2.8. Immunoprecipitation and Western Blot Analyses

To determine whether MnSOD and COX-2 were nitrated, western blot analysis of the immunoprecipitated protein complex and total lysates were performed. In particular, for immunoprecipitation, 300 µg of solubilised proteins, obtained as previously described, were incubated with 10 µg of agarose bead-conjugate, anti-nitrotyrosine, monoclonal antibodies (Upstate Biotechnology) washed in PBS (pH 7.4). The bead–antibodies and binding proteins were resuspended in 50 µL of sample buffer (2×, 0.5 M Tris-HCl (pH 6.8), 2.5% glycerol/0.5% SDS/200 mM 2-mercaptoethanol/0.001% bromophenol blue) and then boiled for 5 min at 95 °C. the immunoprecipitated proteins were resolved in 12% SDS-PAGE mini, and the proteins were transferred to nitrocellulose membranes. The membranes were blocked for 1 h at room temperature in 1% Bovine serum albumin (BSA), 0.1% thimerosal in 50 mM Tris-HCl (pH 7.4), and 150 mM NaCl, 0.01% Tween-20 (TBS/T), followed by incubation with rabbit polyclonal antibodies for MnSOD (O/N, 4 °C, 1:1000; Millipore, code 06984), COX-2 (O/N, 4 °C, 1:500; Cayman Chemical, code 160116), and nitrotyrosine (O/N, 4 °C, 1:1000; Millipore, code AB5411). Membranes were then washed with TBS/T and incubated with a secondary antibody conjugated to horseradish peroxidise (1:15,000; GE Healthcare) for 1 h at room temperature. After washes, the proteins were visualized by enhanced chemiluminescence (ECL; Pierce Biotechnology). No difference in monoclonal β-actin (O/N, 4 °C, 1:5000; Sigma, code A3853) was detected among the lanes. All the densitometry units were normalized against actin for each lane, and are expressed as the ratio of nitrated to unnitrated proteins. Protein bands were quantified by densitometry using Image Quant 5.2 software (Molecular Dynamics).

### 2.9. Determination of MnSOD Activity

MnSOD activity was measured as previously described [1,35,36]. Briefly, the solubilized proteins of paw soft tissue, obtained through cytosolic extraction, were homogenized with 10 mM phosphate-buffered saline (pH 7.4) in a Polytron homogenizer, sonicated on ice for 10 min, and subsequently centrifuged for 10 min at 1.100× *g*.

To determinate MnSOD activity, a competitive inhibition assay that used xanthine–xantine oxidase-generated O_2_ to reduce nitrobluetetrazolium (NTB) to blue tetrazolium salt, was performed as previously described [1,35,36]. The reaction took place in sodium carbonate buffer (50 mM, pH 10.1) containing EDTA (0.1 mM), nitrobluetetrazolium (25 µM), xanthine (0.1 mM), and xanthine oxidase (2 nM). The rate of NTB reduction was monitored at 560 nm. The amount of protein required to inhibit the rate of NTB reduction by 50% was defined as 1 unit of enzyme activity. Enzymatic activity was expressed in units per mg of protein. All determinations were performed in triplicate.

### 2.10. In-Gel Tryptic Digestion

Proteins of paw soft tissue, obtained as previously described through cytosolic extraction, were solubilized in NuPage LDS Sample Buffer (invitrogene), heated at 70 °C for 10 min, separated on precast 4–20% Bis-Tris gels (Invitrogen), and stained with colloidal Coomassie Blue. After visualization, each protein spot was cut and each gel slice was de-stained in 50 mM ammonium hydrogen carbonate/acetonitrile 1:1, and covered with acetonitrile for the reduction of gel pieces. Under a vacuum, centrifugation was performed to remove acetonitrile and to try gel particles. Proteins were suspended in 10 mM dithiothreitol (DTT) and 25 mM (NH_4_)_2_CO_3_ at 56 °C for 30 min, and then cooled and alkylated in 55 mM Iodoacetamide (IAA) and 25 mM (NH_4_)_2_CO_3_ for 30 min in the dark at room temperature. Gel pieces were washed in 50 mM (NH_4_)HCO_3_/CH_3_CN 1:1 for 15 min and covered by acetonitrile until gel pieces shrunk. Under the vacuum, centrifugation allowed acetonitrile removal and gel particles drying. In-gel digestion was performed overnight at 37 °C under stirring, by adding 12.5 ng/μL of trypsin in 25 mM (NH_4_)_2_CO_3_. MALDI mass spectra were recorded directly from the overlay.

### 2.11. MALDI/MS Analysis

Samples (1 μL) were applied to the target and air-dried. Subsequently, 1 μL of α-cyano-4- hydroxycinnamic acid (10 mg/mL) in 50 % acetonitrile and 0.1 % Trifluoroacetic acid (TFA) (*v*/*v*) was applied to the sample and dried again. MALDI mass spectra were recorded using a Voyager-DE STR Applied Biosystems, (United States), a MALDI time-of-flight (MALDI-ToF) mass spectrometer, using the reflectron mode of operation. Ionization was performed with a 337 nm pulsed nitrogen laser. Mass calibration was internally performed using the molecular ions from the trypsin autodigestion. Raw data were analysed using Data Explorer software provided by the manufacturer, and reported as monoisotopic masses. MALDI mass spectra were compared to identify signals with a mass difference of 45 kDa (aminoacidic nitration).

### 2.12. Statistical Analysis

The Kolmogorov–Smirnov test was used for analysis of the data distribution. After confirmation of normal data, differences between groups were compared by analysis of variance (ANOVA). The results are expressed as mean ± SEM. Two-way repeated measures ANOVA with Bonferroni comparisons were used for data obtained from each time point. Other data were analyzed via one-way ANOVA followed by the Newman–Keuls test. The level of statistical significance was fixed at *p* < 0.05. Analyses were carried out using GraphPad Prism software (v8.00; GraphPad Software, Inc.).

## 3. Results

### 3.1. Effects of MnTBAP on Carrageenan-Induced Thermal Hyperalgesia and Oedema

**Figure 1:**
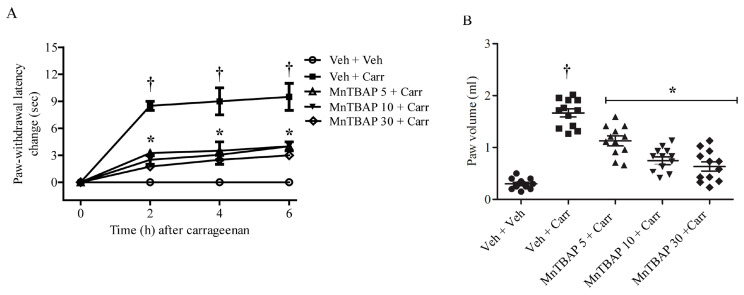
(**A**) Intraplantar injection of carrageenan caused thermal hyperalgesia. This response was blocked by Mn (III) tetrakis (4-benzoic acid) porphyrin (MnTBAP) (5–30 mg/kg). Drugs were given intraperitoneally (i.p.) 15 min before the intraplantar injection of carrageenan. (**B**) The development of thermal hyperalgesia coincided with oedema formation. Injection of different doses of MnTBAP (5–30 mg/kg) was able to reduce oedema. The paw volume (mL) obtained at 6 h after the intraplantar injection of carrageenan. Results are expressed as mean ± SEM for 12 rats. Results are expressed as mean ± SEM for 12 rats. † *p* < 0.05 compared to Veh + Veh; * *p* < 0.05 compared to Veh + Carr.

**Figure 2:**
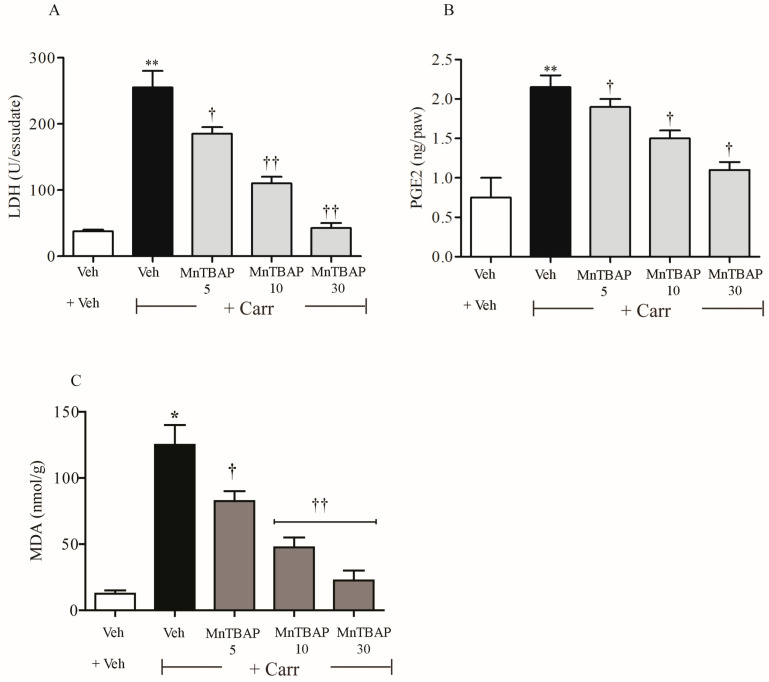
Intraperitoneal injection of MnTBAP in different doses (5–30 mg/kg) led to an improvement in lactate dehydrogenase (LDH) (**A**), prostaglandin E_2_ (PGE_2_) (**B**), and malondialdehyde (MDA) (**C**) levels in paw exudates, observed following the intraplantar injection of carrageenan. Results are expressed as mean ± SEM for six rats. * *p* < 0.01, ** *p* < 0.001 compared with Veh + Veh; † *p* < 0.01, †† *p* < 0.001 compared with Veh + Carr.

Intraplantar (i.pl.) injection of carrageenan in rats determined a time-dependent development of thermal hyperalgesia, which showed up 2 h after the administration of carrageenan (Figure 1A). The development of thermal hyperalgesia coincided with tissue damage and inflammation, as evidenced by oedema (Figure 1B) and supported by previous data [26,28,37]. This condition was associated with LDH, PGE_2_, and MDA accumulation in paw exudates (Figure 2).

Intraperitoneal (i.p.) injection of a different dose of MnTBAP (5 mg/kg, 10 mg/kg, and 30 mg/kg, 15 min before carrageenan) reduced the thermal hyperalgesia and led to a significant improvement in tissue damage and inflammation, characterized by the inhibition of oedema (Figure 1). Furthermore, we observed that the administration of MnTBAP (5–30 mg/kg) led to a reduction of LDH, PGE_2_, and MDA levels in paw exudates (Figure 2), observed after 6 h from carrageenan administration.

### 3.2. Carrageenan-Induced Thermal Hyperalgesia and Oedema were Associated with Nitration and Deactivation of MnSOD

**Figure 3:**
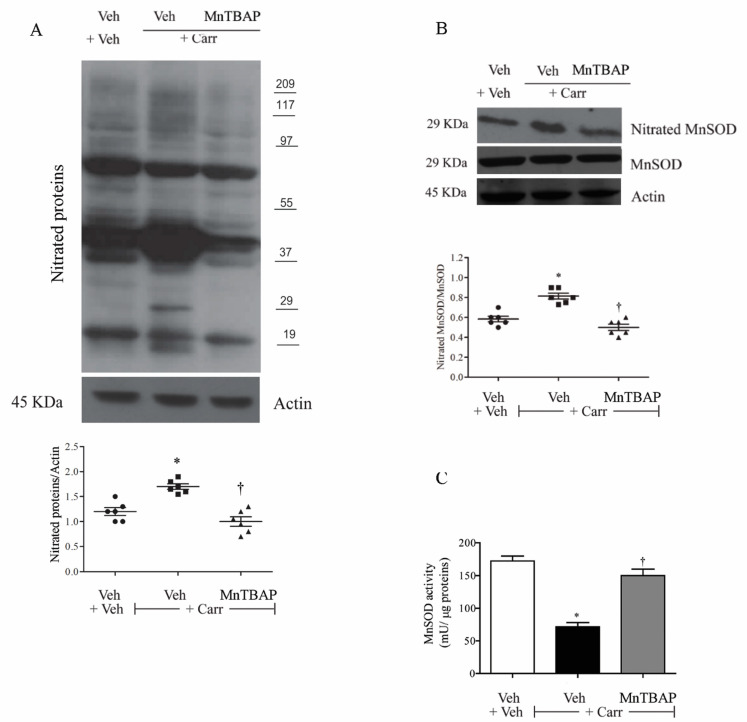
(**A**,**B**) When compared with the vehicle (Veh) group, animals with carrageenan showed nitration of total proteins, including manganese superoxide dismutase (MnSOD). (**C**) Nitration of MnSOD was linked to the deactivation of MnSOD’s enzymatic function. Compared with the vehicle group, animals that received a carrageenan injection showed lower levels of MnSOD activity in the paws. (**A**–**C**) Pretreatment with MnTBAP (10 mg/kg) restored the total proteins and MnSOD nitration and its activity in carrageenan-inflamed rats. No difference in MnSOD or β-actin expression was detected among the lanes in these conditions. The gels are representative of results from six animals, and the histogram represents densitometric analyses of all animals per group. Results are expressed as mean ± SEM for six rats. * *p* < 0.001 compared to Veh + Veh; † *p* < 0.001 compared to Veh + Carr.

The development of thermal hyperalgesia and oedema after intraplantar injection of carrageenan were associated with tyrosine nitration of total proteins (Figure 3A), and in particular of mitochondrial manganese O_2_^−^ dismutase (MnSOD), as evidenced by immunoprecipitation analysis (Figure 3B). Through biochemical analysis, we observed that nitration of MnSOD led to its deactivation, losing its ability to desmute and hence remove superoxide (Figure 3C) [38].

MnTBAP (10 mg/kg) pretreatment (intermediate dose), intraperitoneally administered 15 min before carrageenan, blocked protein nitration (Figure 3A,B) and restored the enzymatic activity of MnSOD (Figure 3B).

### 3.3. Carrageenan-Induced Thermal Hyperalgesia and Oedema were Associated with Nitration of COX-2

**Figure 4:**
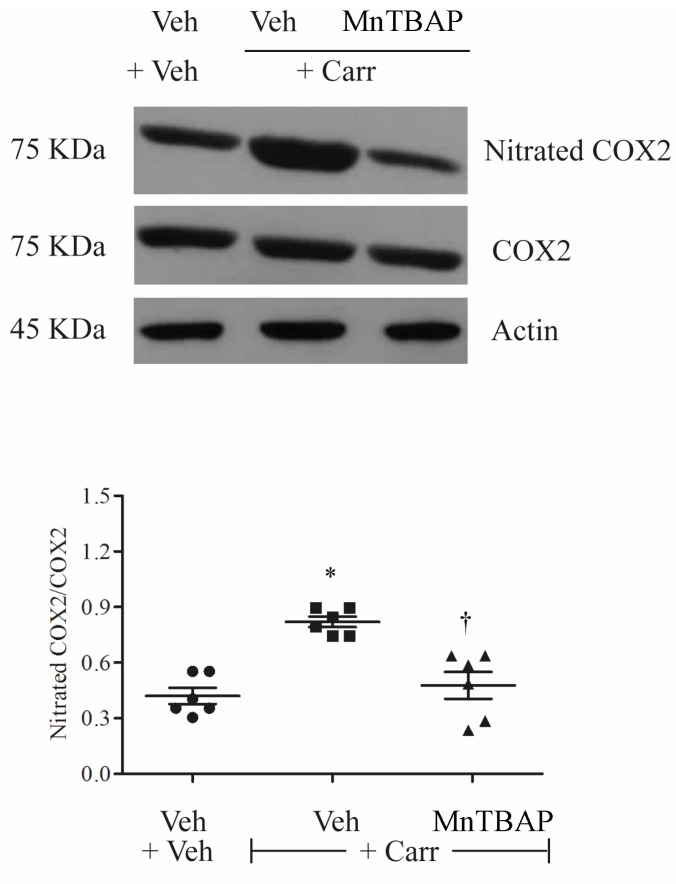
Compared with the vehicle group, animals that received a carrageenan injection showed significant nitration of cyclooxygenase 2 (COX-2). Administration of MnTBAP (10 mg/kg) 15 min before carrageenan partially attenuated COX-2 nitration. No difference for COX-2 or β-actin expression was detected among the lanes in these conditions. Gels are representative of results from six animals, and the histogram represents densitometric analyses of all animals per groups. Results are expressed as mean ± SEM for six rats. * *p* < 0.001 compared to Veh + Veh; † *p* < 0.001 compared to Veh + Carr.

**Figure 5:**
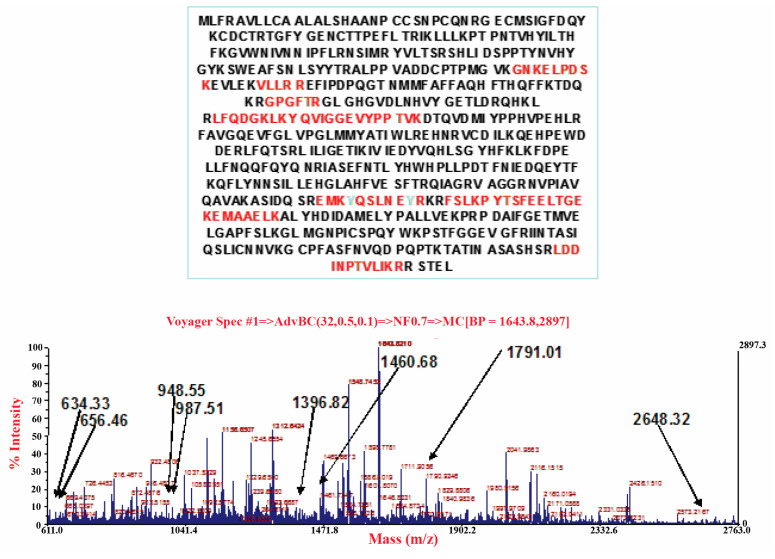
Identification by mass spectrometry of COX-2 after the intraplantar injection of carrageenan.

**Figure 6:**
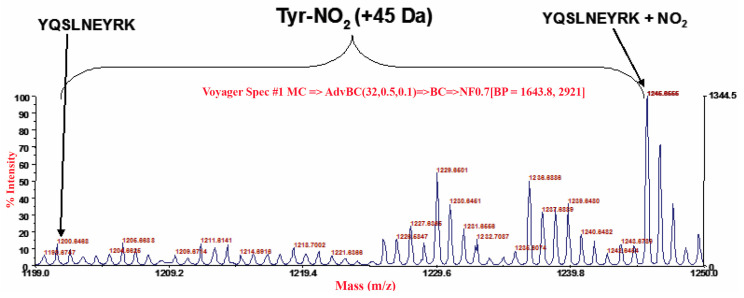
Identification of nitrated tyrosine of COX-2 after the administration of carrageenan.

Intraplantar administration of carrageenan was associated with nitration of tyrosine, not only of the mitochondrial isoform of superoxide dismutase (SOD), but also different proteins expressed during inflammation, including inducible COX-2 in paw tissues, as shown by immunoprecipitation (Figure 4). This enzyme was released at the site of tissue injury and produced high levels of PGE_2_ (Figure 2), a hormone-like substance that stimulates pain and inflammation [39]. Administration of MnTBAP (10 mg/kg) 15 min before carrageenan blocked COX-2 nitration (Figure 4). This data was subsequently confirmed by the analysis of the enzyme in MALDI-TOF, after identification in the mass of COX-2 (Figure 5) was underlined as the tyrosine residues of COX-2 were nitrated (Figure 6). The nitration of the enzyme was well-correlated with the formation of PGE_2_, and with the hyperalgesic responses to carrageenan.

## 4. Discussion

Inflammatory pain is a serious health problem. Its treatment, despite the use of NSAIDs, remains inaccurate, expensive, and sometimes useless. Clinical trials have shown that free radicals, responsible for the onset of oxidative stress, are implicated in the development of numerous diseases, including acute and chronic inflammation [40,41]. Here we show that intraplantar injection of carrageenan leads to the development of thermal hyperalgesia, which is associated with inflammation, as evidenced by oedema, thus reinforcing our previous published data [26,28,37].

High levels of ROS/RNS species and oxidative stress promote the oxidation of biological molecules, including DNA, lipids, and proteins in plasma and mitochondrial membranes [28]. This process leads to the production of lipid peroxidation products, such as 4-hydroxynonenal (4-HNE), malondialdehyde (MDA), and reactive aldehydes [42] as well as to the formation of nitrating species, such as peroxynitrite, nitrosonium cation, nitrogen dioxide, and nitrous peroxocarbonate, promoting the nitration of tyrosine and therefore the inactivation of cellular proteins [43,44].

Our results strongly suggest that during the well-known model of carrageenan-induced inflammation and hyperalgesia in rats, the MDA level increases in association with an increment of lactate dehydrogenase (LDH) and prostaglandin E_2_ (PGE_2_) levels released in paw exudates, thus suggesting their pivotal role in the generation of hyperalgesia and inflammation. These effects were significantly attenuated by pre-treatment with MnTBAP, an inhibitor of lipid peroxidation.

It is well known that prostaglandins (PGs) are the main lipid mediators, with important functions during inflammatory processes. They are synthesized from arachidonic acid by two isoforms of cyclooxygenases, known as COXs (COX-1 or COX-2).

Specifically, COX-1, expressed in many tissues, plays a protective role, providing PG involvement in homeostatic functions [45,46]; COX-2, upregulated by inflammatory mediators, growth factors, and hormones, is an important source of prostanoids during inflammation and leads to robust production of pro-inflammatory PGs [10].

Prostaglandin E_2_ (PGE_2_), the most abundant PG, is involved in many biological functions, but its deregulation or degradation has been associated with several pathological conditions, including the development of acute and chronic inflammation [45,47,48].

In recent years, a different mechanism of PGE_2_ production has been observed in mice and rats during carrageenan-induced inflammatory pain. In particular, it was demonstrated that the weight and mouse’s age are critical elements for oedema and correlated enzyme (COX, PGE_2_) development [49]. Different from rats (where oedema appears during the acute phase of inflammation), a biphasic oedema development has been observed in mice. In fact, 7/8-week-old mice show the first oedema insurgence during the 6 h after carrageenan injection, followed by a second development at 24h [49]. In the same study [49], PGE_2_ production has been observed in the first oedema’s phase (during the first 6 h) due to COX-1, and during the following phase due to COX-2. Conversely, PGE_2_ production in rats is constant during all the inflammatory phase [33,50,51]. These findings underline a more complex COX regulation in mice than in rats.

In addition, previous studies have demonstrated that NO and PGs released during inflammation is responsible of the activation and deliver of an inducible isoform of nitric oxide synthase (iNOS) and COX-2 [20,22,52], which involved in the regulation of several physiological and pathological disease states [52,53].

**Figure 7:**
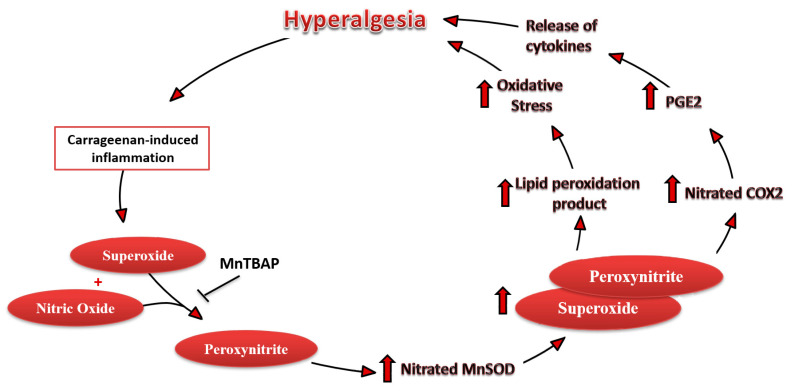
Scheme summarizing the findings of this study. The role of oxidative stress on COX-2 and MnSOD pathways related to the continuous reactive oxygen species (ROS) production for the development and maintenance of the pain state.

In these conditions, tissue inflammation is characterized also by superoxide production that, interacting with NO, leads to the peroxynitrite formation (Figure 7).

Our previous work has already documented the critical role of nitroxidative species, including peroxynitrite, in the development of peripheral and central sensitization associated with pain of diverse etiologies, such as inflammatory and neuropathic pain [1,5,26,27,29,54,55].

In particular, peroxynitrite, a pro-inflammatory and cytotoxic agent, is responsible for protein nitration, including MnSOD, a mitochondrial enzyme able to desmute and remove high levels of superoxide (Figure 7). In the last decade, it has been observed that post-translational modifications of MnSOD are crucial for the continuous formation of free radicals. In fact, when nitrated MnSOD is inactive, it leads to a vicious cycle of free radical formation. This event leads, in turn, to a nitrosative condition, with the nitration of several crucial enzymes involved in the development and maintenance of hyperalgesia and central sensitization, which maintains nociceptive signaling, in pain of different etiologies [21,54,56,57]. In particular, in the present set of experiments, we observed that COX-2 nitration and activation occur in concomitance of MnSOD nitration and deactivation, in the presence of paw oedema and hyperalgesia. Moreover, with the administration of MnTBAP, a peroxynitrite decomposition catalyst, we observed a reduction of these proteins’ nitration and hyperalgesia, most probably due to the peroxynitrite removal.

The excess of free radicals during the pain state is generally inactivated by antioxidants, which inhibit the oxidation of other molecules by directly removing free radicals (pro-oxidants) and providing maximal protection for biological sites [9,37].

It is well-known that antioxidants are classified based on their chemical nature or mode of function [16,58,59,60,61,62]. Our recent studies have reported the use of synthetic antioxidants for the removal of free radicals [6,16,37]. Among these, a metalloporphyrin, known as MnTBAP, is a potent inhibitor of lipid peroxidation and exerts a protective effect associated with various human diseases, most probably due to its peroxynitrite scavenger activity [5]. Indeed, here, we observe that COX-2 undergoing nitration is highly regulated by free radicals and therefore peroxynitrite, resulting in a continuous release of PGs. We also showed that the MnTBAP antioxidant was able to rescue endogenous antioxidant system activity, inactivated during the development of hyperalgesia. Furthermore, antioxidant employment prevented the nitration of MnSOD and COX-2, inhibiting hyperalgesia development during carrageenan injection.

In conclusion, these results reinforce the notion that free radicals and peroxynitrite are important mediators of the induction of hyperalgesia and inflammation. Moreover, peroxynitrite, through the nitration of the tyrosine residue of MnSOD and COX-2, contributes to the formation of free radicals and to the biosynthesis of prostaglandins in rat paw exudates, thus playing a key role as pro-inflammatory and pro-nociceptive agents in a novel mechanism of peripheral sensitization. This mechanism can be pharmacologically restored through the use of MnTBAP, most probably due to its ability to remove peroxynitrite.

In this contest, where the complexity of pain requires new therapeutic approaches, the identification of innovative drugs is crucial to improve patient’s rehabilitation and life quality.

## References

[1] Wang Z.Q., Porreca F., Cuzzocrea S., Galen K., Lightfoot R., Masini E., Muscoli C., Mollace V., Ndengele M., Ischiropoulos H. (2004). A newly identified role for superoxide in inflammatory pain. J. Pharmacol. Exp. Ther..

[2] Maroon J.C., Bost J.W., Borden M.K., Lorenz K.M., Ross N.A. (2006). Natural antiinflammatory agents for pain relief in athletes. Neurosurg. Focus.

[3] Henschke N., Kamper S.J., Maher C.G. (2015). The epidemiology and economic consequences of pain. Mayo Clin. Proc..

[4] Yam M.F., Loh Y.C., Tan C.S., Khadijah Adam S., Abdul Manan N., Basir R. (2018). General Pathways of Pain Sensation and the Major Neurotransmitters Involved in Pain Regulation. Int. J. Mol. Sci..

[5] Muscoli C., Dagostino C., Ilari S., Lauro F., Gliozzi M., Bardhi E., Palma E., Mollace V., Salvemini D. (2013). Posttranslational nitration of tyrosine residues modulates glutamate transmission and contributes to N-methyl-D-aspartate-mediated thermal hyperalgesia. Mediat. Inflamm..

[6] Muscoli C., Cuzzocrea S., Ndengele M.M., Mollace V., Porreca F., Fabrizi F., Esposito E., Masini E., Matuschak G.M., Salvemini D. (2007). Therapeutic manipulation of peroxynitrite attenuates the development of opiate-induced antinociceptive tolerance in mice. J. Clin. Investig..

[7] Watkins L.R., Milligan E.D., Maier S.F. (2001). Spinal cord glia: New players in pain. Pain.

[8] Yamacita-Borin F.Y., Zarpelon A.C., Pinho-Ribeiro F.A., Fattori V., Alves-Filho J.C., Cunha F.Q., Cunha T.M., Casagrande R., Verri W.A. (2015). Superoxide anion-induced pain and inflammation depends on TNFalpha/TNFR1 signaling in mice. Neurosci. Lett..

[9] Nistico S., Ventrice D., Dagostino C., Lauro F., Ilari S., Gliozzi M., Colica C., Musolino V., Carresi C., Strongoli M.C. (2013). Effect of MN (III) tetrakis (4-benzoic acid) porphyrin by photodynamically generated free radicals on SODs keratinocytes. J. Biol. Regul. Homeost. Agents.

[10] Mollace V., Muscoli C., Masini E., Cuzzocrea S., Salvemini D. (2005). Modulation of prostaglandin biosynthesis by nitric oxide and nitric oxide donors. Pharmacol. Rev..

[11] Salvemini D., Doyle T.M., Cuzzocrea S. (2006). Superoxide, peroxynitrite and oxidative/nitrative stress in inflammation. Biochem. Soc. Trans..

[12] Cuzzocrea S., Salvemini D. (2007). Molecular mechanisms involved in the reciprocal regulation of cyclooxygenase and nitric oxide synthase enzymes. Kidney Int..

[13] Liu Z., Ren Z., Zhang J., Chuang C.C., Kandaswamy E., Zhou T., Zuo L. (2018). Role of ROS and Nutritional Antioxidants in Human Diseases. Front. Physiol..

[14] Salvemini D., Riley D.P., Lennon P.J., Wang Z.Q., Currie M.G., Macarthur H., Misko T.P. (1999). Protective effects of a superoxide dismutase mimetic and peroxynitrite decomposition catalysts in endotoxin-induced intestinal damage. Br. J. Pharmacol..

[15] Muscoli C., Doyle T., Dagostino C., Bryant L., Chen Z., Watkins L.R., Ryerse J., Bieberich E., Neumman W., Salvemini D. (2010). Counter-regulation of opioid analgesia by glial-derived bioactive sphingolipids. J. Neurosci..

[16] Muscoli C., Cuzzocrea S., Riley D.P., Zweier J.L., Thiemermann C., Wang Z.Q., Salvemini D. (2003). On the selectivity of superoxide dismutase mimetics and its importance in pharmacological studies. Br. J. Pharmacol..

[17] Obrosova I.G., Julius U.A. (2005). Role for poly(ADP-ribose) polymerase activation in diabetic nephropathy, neuropathy and retinopathy. Curr. Vasc. Pharmacol..

[18] Kauppinen A., Suuronen T., Ojala J., Kaarniranta K., Salminen A. (2013). Antagonistic crosstalk between NF-kappaB and SIRT1 in the regulation of inflammation and metabolic disorders. Cell Signal.

[19] Komirishetty P., Areti A., Gogoi R., Sistla R., Kumar A. (2017). Combination strategy of PARP inhibitor with antioxidant prevent bioenergetic deficits and inflammatory changes in CCI-induced neuropathy. Neuropharmacology.

[20] Salvemini D., Kim S.F., Mollace V. (2013). Reciprocal regulation of the nitric oxide and cyclooxygenase pathway in pathophysiology: Relevance and clinical implications. Am. J. Physiol. Regul. Integr. Comp. Physiol..

[21] Doyle T., Chen Z., Muscoli C., Obeid L.M., Salvemini D. (2011). Intraplantar-injected ceramide in rats induces hyperalgesia through an NF-kappaB- and p38 kinase-dependent cyclooxygenase 2/prostaglandin E2 pathway. FASEB J..

[22] Sorokin A. (2016). Nitric Oxide Synthase and Cyclooxygenase Pathways: A Complex Interplay in Cellular Signaling. Curr. Med. Chem..

[23] Korbecki J., Baranowska-Bosiacka I., Gutowska I., Chlubek D. (2013). The effect of reactive oxygen species on the synthesis of prostanoids from arachidonic acid. J. Physiol. Pharmacol..

[24] Grosser T., Theken K.N., FitzGerald G.A. (2017). Cyclooxygenase Inhibition: Pain, Inflammation, and the Cardiovascular System. Clin. Pharmacol. Ther..

[25] Lauro F., Giancotti L.A., Ilari S., Dagostino C., Gliozzi M., Morabito C., Malafoglia V., Raffaeli W., Muraca M., Goffredo B.M. (2016). Inhibition of Spinal Oxidative Stress by Bergamot Polyphenolic Fraction Attenuates the Development of Morphine Induced Tolerance and Hyperalgesia in Mice. PLoS ONE.

[26] Lauro F., Ilari S., Giancotti L.A., Ventura C.A., Morabito C., Gliozzi M., Malafoglia V., Palma E., Paolino D., Mollace V. (2016). Pharmacological effect of a new idebenone formulation in a model of carrageenan-induced inflammatory pain. Pharmacol. Res..

[27] Muscoli C., Lauro F., Dagostino C., Ilari S., Giancotti L.A., Gliozzi M., Costa N., Carresi C., Musolino V., Casale F. (2014). Olea Europea-derived phenolic products attenuate antinociceptive morphine tolerance: An innovative strategic approach to treat cancer pain. J. Biol. Regul. Homeost. Agents.

[28] Ilari S., Giancotti L.A., Lauro F., Dagostino C., Gliozzi M., Malafoglia V., Sansone L., Palma E., Tafani M., Russo M.A. (2020). Antioxidant modulation of sirtuin 3 during acute inflammatory pain: The ROS control. Pharmacol. Res..

[29] Little J.W., Doyle T., Salvemini D. (2012). Reactive nitroxidative species and nociceptive processing: Determining the roles for nitric oxide, superoxide, and peroxynitrite in pain. Amino Acids.

[30] (1980). Ethical standards for investigations of experimental pain in animals. The Committee for Research and Ethical Issues of the International Association for the Study of Pain. Pain.

[31] Batinic-Haberle I., Cuzzocrea S., Reboucas J.S., Ferrer-Sueta G., Mazzon E., Di Paola R., Radi R., Spasojevic I., Benov L., Salvemini D. (2009). Pure MnTBAP selectively scavenges peroxynitrite over superoxide: Comparison of pure and commercial MnTBAP samples to MnTE-2-PyP in two models of oxidative stress injury, an SOD-specific Escherichia coli model and carrageenan-induced pleurisy. Free Radic. Biol. Med..

[32] Ndengele M.M., Cuzzocrea S., Esposito E., Mazzon E., Di Paola R., Matuschak G.M., Salvemini D. (2008). Cyclooxygenases 1 and 2 contribute to peroxynitrite-mediated inflammatory pain hypersensitivity. FASEB J..

[33] Salvemini D., Wang Z.Q., Wyatt P.S., Bourdon D.M., Marino M.H., Manning P.T., Currie M.G. (1996). Nitric oxide: A key mediator in the early and late phase of carrageenan-induced rat paw inflammation. Br. J. Pharmacol..

[34] Hargreaves K., Dubner R., Brown F., Flores C., Joris J. (1988). A new and sensitive method for measuring thermal nociception in cutaneous hyperalgesia. Pain.

[35] Beauchamp C., Fridovich I. (1971). Superoxide dismutase: Improved assays and an assay applicable to acrylamide gels. Anal. Biochem..

[36] Nishida S., Teramoto K., Kimoto-Kinoshita S., Tohda Y., Nakajima S., Tomura T.T., Irimajiri K. (2002). Change of Cu,Zn-superoxide dismutase activity of guinea pig lung in experimental asthma. Free Radic. Res..

[37] Mollace V., Muscoli C., Dagostino C., Giancotti L.A., Gliozzi M., Sacco I., Visalli V., Gratteri S., Palma E., Malara N. (2014). The effect of peroxynitrite decomposition catalyst MnTBAP on aldehyde dehydrogenase-2 nitration by organic nitrates: Role in nitrate tolerance. Pharmacol. Res..

[38] Doyle T., Bryant L., Batinic-Haberle I., Little J., Cuzzocrea S., Masini E., Spasojevic I., Salvemini D. (2009). Supraspinal inactivation of mitochondrial superoxide dismutase is a source of peroxynitrite in the development of morphine antinociceptive tolerance. Neuroscience.

[39] Desai S.J., Prickril B., Rasooly A. (2018). Mechanisms of Phytonutrient Modulation of Cyclooxygenase-2 (COX-2) and Inflammation Related to Cancer. Nutr. Cancer.

[40] Hussain T., Tan B., Yin Y., Blachier F., Tossou M.C., Rahu N. (2016). Oxidative Stress and Inflammation: What Polyphenols Can Do for Us?. Oxid. Med. Cell Longev..

[41] Grace P.M., Gaudet A.D., Staikopoulos V., Maier S.F., Hutchinson M.R., Salvemini D., Watkins L.R. (2016). Nitroxidative Signaling Mechanisms in Pathological Pain. Trends Neurosci..

[42] McGarry T., Biniecka M., Veale D.J., Fearon U. (2018). Hypoxia, oxidative stress and inflammation. Free Radic. Biol. Med..

[43] Radi R. (2013). Protein tyrosine nitration: Biochemical mechanisms and structural basis of functional effects. Acc. Chem. Res..

[44] Janes K., Neumann W.L., Salvemini D. (2012). Anti-superoxide and anti-peroxynitrite strategies in pain suppression. Bba-Mol. Basis Dis..

[45] Ricciotti E., FitzGerald G.A. (2011). Prostaglandins and inflammation. Arterioscler. Thromb. Vasc. Biol..

[46] Astakhova A., Chistyakov D., Thomas D., Geisslinger G., Brune B., Sergeeva M., Namgaladze D. (2019). Inhibitors of Oxidative Phosphorylation Modulate Astrocyte Inflammatory Responses through AMPK-Dependent Ptgs2 mRNA Stabilization. Cells.

[47] Gadek-Michalska A., Tadeusz J., Rachwalska P., Bugajski J. (2013). Cytokines, prostaglandins and nitric oxide in the regulation of stress-response systems. Pharmacol. Rep..

[48] Kim S.F. (2014). The nitric oxide-mediated regulation of prostaglandin signaling in medicine. Vitam. Horm..

[49] Posadas I., Bucci M., Roviezzo F., Rossi A., Parente L., Sautebin L., Cirino G. (2004). Carrageenan-induced mouse paw oedema is biphasic, age-weight dependent and displays differential nitric oxide cyclooxygenase-2 expression. Br. J. Pharmacol..

[50] Handy R.L., Moore P.K. (1998). A comparison of the effects of L-NAME, 7-NI and L-NIL on carrageenan-induced hindpaw oedema and NOS activity. Br. J. Pharmacol..

[51] Omote K., Hazama K., Kawamata T., Kawamata M., Nakayaka Y., Toriyabe M., Namiki A. (2001). Peripheral nitric oxide in carrageenan-induced inflammation. Brain Res..

[52] Segelcke D., Reichl S., Neuffer S., Zapp S., Ruther T., Evers D., Zahn P.K., Pogatzki-Zahn E.M. (2020). The role of the spinal cyclooxygenase (COX) for incisional pain in rats at different developmental stages. Eur. J. Pain.

[53] de Paula T.D., Silva B.R., Grando M.D., Pernomian L., do Prado A.F., Bendhack L.M. (2017). Relaxation induced by the nitric oxide donor and cyclooxygenase inhibitor NCX2121 in renal hypertensive rat aortas. Eur. J. Pharm. Sci..

[54] Muscoli C., Mollace V., Wheatley J., Masini E., Ndengele M., Wang Z.Q., Salvemini D. (2004). Superoxide-mediated nitration of spinal manganese superoxide dismutase: A novel pathway in N-methyl-D-aspartate-mediated hyperalgesia. Pain.

[55] Salvemini D., Little J.W., Doyle T., Neumann W.L. (2011). Roles of reactive oxygen and nitrogen species in pain. Free Radic. Biol. Med..

[56] Chen Z., Muscoli C., Doyle T., Bryant L., Cuzzocrea S., Mollace V., Mastroianni R., Masini E., Salvemini D. (2010). NMDA-receptor activation and nitroxidative regulation of the glutamatergic pathway during nociceptive processing. Pain.

[57] Doyle T., Bryant L., Muscoli C., Cuzzocrea S., Esposito E., Chen Z., Salvemini D. (2010). Spinal NADPH oxidase is a source of superoxide in the development of morphine-induced hyperalgesia and antinociceptive tolerance. Neurosci. Lett..

[58] Birben E., Sahiner U.M., Sackesen C., Erzurum S., Kalayci O. (2012). Oxidative stress and antioxidant defense. World Allergy Organ. J..

[59] Abdelhalim M.A.K., Moussa S.A.A., Qaid H.A., Al-Ayed M.S. (2018). Potential effects of different natural antioxidants on inflammatory damage and oxidative-mediated hepatotoxicity induced by gold nanoparticles. Int. J. Nanomed..

[60] Blokhina O., Virolainen E., Fagerstedt K.V. (2003). Antioxidants, oxidative damage and oxygen deprivation stress: A review. Ann. Bot..

[61] Bose K.S., Vyas P., Singh M. (2012). Plasma non-enzymatic antioxidants-vitamin C, E, beta-carotenes, reduced glutathione levels and total antioxidant activity in oral sub mucous fibrosis. Eur. Rev. Med. Pharmacol. Sci..

[62] Dudek H., Farbiszewski R., Rydzewska M., Michno T., Kozlowski A. (2004). Evaluation of antioxidant enzymes activity and concentration of non-enzymatic antioxidants in human brain tumours. Wiad. Lek..

